# Acceptability of Parental Financial Incentives and Quasi-Mandatory Interventions for Preschool Vaccinations: Triangulation of Findings from Three Linked Studies

**DOI:** 10.1371/journal.pone.0156843

**Published:** 2016-06-02

**Authors:** Jean Adams, Rebekah J. McNaughton, Sarah Wigham, Darren Flynn, Laura Ternent, Janet Shucksmith

**Affiliations:** 1 MRC Epidemiology Unit, University of Cambridge, Cambridge, United Kingdom; 2 School of Health & Social Care, Teesside University, Middlesbrough, United Kingdom; 3 Institute of Neuroscience, Newcastle University, Newcastle upon Tyne, United Kingdom; 4 Institute of Health & Society, Newcastle University, Newcastle upon Tyne, United Kingdom; University of Waterloo, CANADA

## Abstract

**Background:**

Childhood vaccinations are a core component of public health programmes globally. Recent measles outbreaks in the UK and USA have prompted debates about new ways to increase uptake of childhood vaccinations. Parental financial incentives and quasi-mandatory interventions (e.g. restricting entry to educational settings to fully vaccinated children) have been successfully used to increase uptake of childhood vaccinations in developing countries, but there is limited evidence of effectiveness in developed countries. Even if confirmed to be effective, widespread implementation of these interventions is dependent on acceptability to parents, professionals and other stakeholders.

**Methods:**

We conducted a systematic review (n = 11 studies included), a qualitative study with parents (n = 91) and relevant professionals (n = 24), and an on-line survey with embedded discrete choice experiment with parents (n = 521) exploring acceptability of parental financial incentives and quasi-mandatory interventions for preschool vaccinations. Here we use Triangulation Protocol to synthesise findings from the three studies.

**Results:**

There was a consistent recognition that incentives and quasi-mandatory interventions could be effective, particularly in more disadvantaged groups. Universal incentives were consistently preferred to targeted ones, but relative preferences for quasi-mandatory interventions and universal incentives varied between studies. The qualitative work revealed a consistent belief that financial incentives were not considered an appropriate motivation for vaccinating children. The costs of financial incentive interventions appeared particularly salient and there were consistent concerns in the qualitative work that incentives did not represent the best use of resources for promoting preschool vaccinations. Various suggestions for improving delivery of the current UK vaccination programme as an alternative to incentives and quasi-mandates were made.

**Conclusions:**

Parental financial incentives and quasi-mandatory interventions for increasing uptake of preschool vaccinations do not currently attract widespread enthusiastic support in the UK; but some potential benefits of these approaches are recognised.

## Introduction

Childhood vaccinations are a core component of public health programmes around the world.[1] Despite high vaccination coverage rates in many countries,[2] recent measles outbreaks in the UK [3] and USA [4] have returned childhood vaccination programmes to public attention and prompted debates about new ways to increase uptake.

Structural public health interventions are those which reduce or eliminate individual choice about whether or not to engage with an intervention.[5] These interventions are often considered politically and publically controversial,[6] and potentially unethical.[7] In the case of vaccine-preventable infectious diseases, where the immediate population health consequences of not acting can be significant, such structural interventions may be considered appropriate.[7]

Health promoting financial incentives have been previously defined as “cash or cash-like rewards (e.g. vouchers that can be exchanged for goods or services) or penalties (e.g. reductions in welfare benefits), provided contingent on performance of healthy behaviours” (p2).[8] Financial incentives reduce individual choice to engage with an intervention, by increasing the financial consequences of not engaging.[7] Furthermore, by providing an immediate reward for a behaviour that can be unrewarding in the short-term, financial incentives can work with the common preference for short-, versus long-, term rewards.[9]

Financial incentives have been successfully used to increase uptake of childhood vaccinations in developing countries, and adult vaccinations in developed countries.[8, 10] Providing financial incentives for health behaviours in general has been criticised as coercive and socially divisive.[11] However, recent work has found that these interventions can be acceptable if the problems addressed are perceived to be serious, other interventions are perceived to be ineffective, and incentives confirmed to be both effective and cost-effective.[12–15] Little work has focused specifically on the acceptability of parental financial incentives for increasing uptake of childhood vaccinations.[16] As well as personal health benefits to the recipient, vaccinations also convey a benefit to the wider community by contributing to herd immunity. This makes vaccinations unlike many other health behaviours, where it is generally assumed that only those who take part in healthy behaviours benefit from them. Findings concerning the acceptability of financial incentives in relation to other health behaviours may not, therefore, be transferrable to vaccinations.

Mandating that only fully vaccinated children can attend child-care or school is another structural intervention for promoting uptake of vaccinations. In most cases where this has been implemented, parents can apply for exemptions for medical, philosophical or religious reasons, meaning that such interventions are only ‘quasi-mandatory’. There is some evidence that quasi-mandatory vaccination policies are effective in some cases, but little is known about the acceptability of these interventions.[16]

Acceptability of public health interventions should be considered from the viewpoint of a number of stakeholder groups. These include the target population, professionals involved with intervention delivery, and policy makers responsible for intervention implementation. In order for any health promoting intervention to be effective in practice, members of all stakeholder groups must be both willing and able to engage with it.[17]

We conducted a series of three linked studies exploring the acceptability of parental incentives and quasi-mandatory interventions for increasing uptake of preschool vaccinations in the UK. Neither policy is currently implemented anywhere in the UK. These studies were: a systematic review,[16] a qualitative interview study with parents and a range of relevant professionals,[18] and an on-line survey with an embedded discrete choice experiment (DCE) with parents who did and did not have characteristics associated with incompletely vaccinating their children (Flynn et al., under review). These studies have been reported as stand-alone pieces of work. However, they were conceived as an integrated programme.[19] Specifically, examples of incentive and quasi-mandatory programmes identified in the systematic review were used as discussion prompts in the qualitative study; and early themes identified in the qualitative study were used to guide development of the DCE.

Although the results of each individual study provide useful insights in their own right, together the results of the full programme showed both convergence and divergence, which opened up new debates about the implications of the work. Here we use Triangulation Protocol [20] to draw out wider learning from the combined programme. Triangulation Protocol is a systematic approach to ‘triangulation’ described in more detail below. In general, triangulation involves exploring the convergence, complementarity and dissonance of results on related research questions obtained from different methodological approaches, sources, theoretical perspectives, or researchers. It has been proposed that the validity of conclusions is enhanced if different approaches produce convergent findings.[21]

## Methods

### Primary studies

The primary studies referred to in this paper have been reported in full elsewhere.[16, 18, 22] The research questions, inclusion criteria and sample size of each of the primary studies are summarised in Table 1; the results are summarised here to provide context.

**Table 1:** Summary of study designs, research questions, inclusion criteria and sample size in the three components studies.

	Systematic review	Qualitative study	Discrete choice experiment
Study design	Systematic review and narrative synthesis, with effectiveness, acceptability and economic components.	Focus group interviews with parents of preschool children. Individual interviews with a range of health and other relevant professionals.	On-line survey with questions on participant characteristics, attitudes to and experiences of vaccination; and choice sets exploring preferences for preschool vaccination programmes according to eight attributes, including an incentive.
Research questions	What is the existing evidence on parental incentive and quasi-mandatory schemes for increasing uptake of vaccinations in preschool children in high income countries, compared to usual care or no intervention in terms of: effectiveness, acceptability and economic costs and consequences?	What are stakeholders’ views, wants and needs concerning interventions to promote uptake of preschool vaccination programmes? Would parental incentive or quasi-mandatory schemes for encouraging uptake of preschool vaccinations be viewed as acceptable? Why? What, if anything, could be done to increase acceptability?	What is the value parents place on key attributes and associated attribute levels of preschool vaccination programmes?
Inclusion criteria	The effectiveness component included studies that compared the effects on uptake of preschool vaccinations of included interventions compared to usual care or no intervention using a controlled trial or time series analysis. The acceptability component included studies that explored acceptability of included interventions in any stakeholder group using any study design. The economic component included studies in either the effectiveness or acceptability component that explored economic costs and consequences of interventions.	Parents and carers of preschool children living in the North East of England, recruited from Children’s Centres and baby and toddler groups in localities with high and low levels of deprivation, and which had and had not experienced recent cases or outbreaks of measles. Health and other relevant professionals working in the North East of England.	Parents or guardians of one or more children <5 years old, currently residing in England, and members of an on-line panel held by the sub-contracting market research company. Respondents were stratified according to whether they met any criteria associated with low vaccination: live the 20% most deprived areas of England, have a child <5 years old with a physical or mental disability, are a single parent, are aged less than 20 years, or have more than 3 children.
Sample size	4 studies in the effectiveness component. 6 studies in the acceptability component. 1 study in the economic component.	91 parents or carers in 10 focus groups. 24 health and other professionals, including vaccination policymakers and commissioners (n = 6), GPs and practices nurses (n = 9), health visitors (n = 4), school nurses (n = 1), community paediatricians (n = 2), and primary school head teachers (n = 2).	259 parents with characteristics associated with low vaccination. 262 parents without characteristics associated with low vaccination.

#### Systematic review [16]

The systematic review identified a number of ways in which financial incentives and quasi-mandatory interventions have been implemented for preschool vaccinations. These were: rewards, paid to all parents, when their children’s vaccinations were complete (universal reward); rewards, offered only to parents whose children have not received all vaccinations, on completion of the vaccination schedule (targeted reward); universal child support payments only paid to the parents of children who are up to date with vaccinations (universal penalty); and entry to child-care or school only available to children who are up to date with vaccinations (quasi-mandatory policy). The review concluded that there was insufficient evidence to draw firm conclusions on the effectiveness or economic costs and consequences of parental incentives or quasi-mandatory interventions for preschool vaccinations.

There was some evidence that quasi-mandatory interventions were more acceptable to parents than parental incentives, but this evidence tended to come from contexts where quasi-mandatory policies were already in place. This reflects research from elsewhere that indicates that acceptability of public health interventions is influenced by familiarity with the intervention.[23]

#### Qualitative study [18]

In the qualitative study, parents and professionals recognised that financial incentives might particularly encourage families who were living in disadvantaged circumstances to prioritise vaccination. However, this benefit could be outweighed by the unintended consequences of turning a behaviour that is generally willingly engaged in, out of a sense of altruism and social responsibility, into a cash transaction. For this reason, both groups felt that offering parents cash payments for vaccinating their children was inappropriate. Financial incentives were also commonly interpreted as ‘bribes’. Given the controversy over the measles, mumps and rubella vaccination in the UK in the 1990s,[24] many viewed this sort of ‘bribe’ as sending a message that there was something inherently ‘wrong’ with preschool vaccinations that only a financial incentive could overcome.

Penalties reducing universal social welfare payments were seen as superficially more attractive than financial rewards by parents. However, parents acknowledged that the most disadvantaged families were very reliant on these payments and that such a policy might inappropriately penalise children for their parents’ decisions. Overall, universal financial rewards were viewed as preferable to those targeted at any particular group (e.g. those who had not had their children vaccinated by a certain age).

The idea of a quasi-mandatory scheme was met with mixed opinions. For many, it seemed like an appropriate option that was fair, equitable and even ‘normal’. Many UK daycare centres and schools already ask about children’s vaccination status to allow them to identify at-risk children during outbreaks. Various other screening and monitoring programmes already run in UK schools. However, refusing children education based on parental vaccination decisions seemed immoral to some parents. For this reason participants believed there would have to be robust procedures in place for parents to legitimately opt-out of vaccinations, for medical or religious reasons. Discussion of incentive and quasi-mandatory schemes consistently returned to the need to strengthen existing programmes via better information provision, professional support and more flexible vaccination delivery.

#### On-line survey with an embedded discrete choice experiment (Flynn et al., under review)

Discrete choice experiments describe interventions according to their key characteristics, or ‘attributes’ (e.g. type of reward, value of incentive), and ‘levels’ of these attributes (e.g. cash, shopping voucher; higher, lower values). Participants are then asked which of a small number of intervention ‘scenarios’, combining different levels of each attribute, they prefer. This allows relative preferences for attribute levels to be determined. Discrete choice experiments are well-established in health economics [25–27] and increasingly used in public health.[14, 28] The DCE was embedded in a wider on-line survey asking questions about general preferences and socio-demographic circumstances.

Respondents to the DCE demonstrated a strong preference for vaccinating their children. Parents had significant preferences for the way in which vaccination services are delivered in terms of staff type, location, expected waiting times and information provision. In terms of financial incentives, there was a general preference for cash rewards, compared to shopping voucher rewards, particularly among parents with characteristics associated with incomplete vaccination. Higher value and universal incentives were preferred to those targeted at particular sub-groups. In a preference elicitation task in the wider survey, most support was given to universal financial rewards, followed by quasi-mandatory interventions, current practice (i.e. no incentive or mandate), and finally targeted financial rewards. Amongst parents who stated that they would require a financial reward to vaccinate their children (n = 122, 25%; but 31% of those with characteristics associated with incomplete vaccination), the average minimum value required was around £110 (~US$159; €147). The average maximum incentive participants believed should be provided, amongst those who stated that they did not require a financial incentive to vaccinate their children, was around £70 (~US$101; €93).

### Triangulation and integration

Four types of triangulation have been described: methodological triangulation where more than one methodological approach is used to collect data; data triangulation where data is collected from more than one data source or respondent group; investigator triangulation where two or more researchers take part in integrative analysis; and theoretical triangulation where different theoretical perspectives or interpretative frameworks are adopted.[21]

We made use of all four of these types of triangulation. A range of both quantitative (DCE, survey and systematic searching in the systematic review) and qualitative (focus groups with parents and carers, individual interviews with health and other professionals, and narrative synthesis in the systematic review) methods were used. This allows methodological triangulation. As data was collected from more than one participant group (see Table 1) data triangulation was possible. As described below, a number of researchers took part in triangulation, allowing investigator triangulation. Finally, the different methods used across the studies drew on different theoretical perspectives—the systematic review, DCE and survey drew on the positivist theoretical perspective, whilst the focus groups and individual interviews drew on the interpretivist theoretical perspective. This means that data collected within different research paradigms are included and provides the opportunity for theoretical triangulation. To some extent, this overlaps with methodological triangulation. Data collected within these different paradigms are integrated during triangulation without any particular preference or primacy given to any particular methodology or theoretical perspective.

We base our approach to triangulation on ‘Triangulation Protocol’.[20] This involves identifying themes from each data source and method, and then sorting these into similar categories. These are then ‘convergence coded’ to identify where there is agreement, dissonance and silence (i.e. where issues identified in one component are not covered in another) in terms of data from different sources and methods. For this exercise, we divided the qualitative study into two components—results from parents and carers; and results from health and other relevant professionals. Similarly, the on-line survey in which the DCE was embedded was split into two components—results from the formal DCE; and results from the wider survey. Initially, convergence coding was conducted by JA. Preliminary results were then discussed amongst the full research team and the convergence coding refined, based on these discussions.

Here we present the results of the convergence coding and highlight and discuss key areas of agreement and apparent contradiction. Our intention is not to repeat the findings from the individual primary studies, and the results presented here do not represent the ‘last word’ on the acceptability of financial incentives and quasi-mandatory interventions for increasing preschool vaccinations—substantial additional information is presented in the descriptions of the primary studies. Instead we focus on what can be learnt from viewing the component studies together, rather than as individual pieces of work. Thus any findings that were apparent from any of the individual component studies alone are not repeated here.

Given the nature of the work, we both report and interpret results in the ‘results’ section to provide an integrated consideration of findings across the three linked primary studies. The discussion section provides a summary of the results, and consideration of the strengths and weaknesses of the method used.

### Research ethics

This work was a secondary analysis of extant data. Ethical approval was not required for this secondary analysis. Ethical approval for the original qualitative study was provided by Teesside University’s School of Health and Social Care Research Ethics and Governance Committee. Ethical approval for the original survey and embedded DCE was provided by Newcastle University’s Faculty of Medicine’s Research Ethics Committee. All personally identifying information was anonymised and de-identified prior to analysis in the primary studies.

## Results and Interpretation

Table 2 shows a summary of the main themes identified in the research, sorted into three overall groups (financial incentives and penalties, quasi-mandatory interventions, and alternative interventions), and ordered to bring related themes near to each other.

**Table 2:** Summary of themes identified in the research, with agreement between research components identified.

Theme	Sys. review	Qual: parents	Qual: professionals	DCE	Questio-nnaire
**Financial incentives & penalties**					
Financial incentives have been successful in some circumstances to encourage healthy behaviours	A^a^	S^b^	A	S	S
~25% of participants would require a financial incentive to vaccinate their children	S	S	S	S	A
Financial incentives could encourage parents experiencing financial hardship to vaccinate	S	A	S	S	S
Universal financial incentives are more equitable than/preferred to targeted incentives	S	A	S	A	A
Targeted financial incentives could lead to parents ‘gaming the system’ and delaying vaccination to become eligible	S	A	S	S	S
Financial penalties are more acceptable than financial rewards	S	A	S	S	S
Financial penalties could act as a timely reminder to vaccinate a child	S	A	S	S	S
Financial incentives are a bribe for being a responsible parent & may break the bonds of social responsibility	S	A	A	S	S
Financial incentives may not be the most efficient use of resources	S	A	A	S	S
Financial incentives would not change the mind of parents who have made a conscious decision not to vaccinate	S	A	S	S	S
Cash rewards are preferable to vouchers	S	S	S	A	S
Higher value rewards are preferable	S	S	S	A	S
**Quasi-mandatory interventions**					
Quasi-mandatory interventions are more acceptable than any type of financial incentives	A	A	A	S	S
Quasi-mandatory interventions are preferable to universal, but not targeted, financial incentives	S	S	S	S	A
Quasi-mandatory interventions offer protection for all children and staff in a shared setting	S	A	S	S	S
Quasi-mandatory interventions would act as a reminder to vaccinate	S	A	S	S	S
Quasi-mandatory interventions would punish children for a decision made by their parent	S	A	S	S	S
Quasi-mandatory interventions remove valued choice to engage with a health-related behaviour	S	A	A	S	S
Quasi-mandatory interventions would have to incorporate clear opt-out processes	S	A	S	S	S
Quasi-mandatory interventions could normalise vaccination	S	S	A	S	S
School entry is an ideal time to monitor vaccination status and provide catch-up vaccinations	S	S	A	S	S
Schools should not become responsible for administration of a quasi-mandatory intervention	S	S	A	S	S
**Alternative interventions to increase vaccination uptake**					
More flexibility is required in the timing and location of where vaccinations are delivered, with less waiting time	S	A	A	A	S
Information & education about vaccination and related diseases needs to be more accessible to parents	S	A	A	S	S
Information on risks & benefits provided in numerical format is preferable to that in chart or pictorial format	S	S	S	A	S
Professionals must build trusting relationships with parents and listen to their fears	S	S	A	S	S
Better multi-disciplinary working and information sharing is required	S	S	A	S	S
Vaccinations provided by pharmacists are less preferred than those provided by practice nurse at GP surgery	S	S	S	A	S
Vaccinations provided by community nurses in a mobile bus are less preferred those provided by practice nurse at GP surgery	S	S	S	A	S

Sys. Review: systematic review; Qual.–parents: qualitative study with parents and carers; Qual.–professionals: qualitative study with health and other relevant professionals; DCE: discrete choice experiment; Survey: questionnaire included with DCE;

^a^A (agreement) indicates that a theme was present in results from a research component,

^b^S (silence) indicates that a theme was absent in results from a research component.

In Table 2, As (agreement) and Ss (silence) indicate whether a theme was identified, or not, in a particular research component. In most cases, silence reflects differences in the research questions across studies (see Table 1). We did not identify any clear instances of dissonance with disagreement on a theme between research components. However, there are themes that could be interpreted as potentially contradictory. These are discussed further below.

### Potential and perceived effectiveness of parental financial interventions

The systematic review identified that financial incentives and quasi-mandatory interventions have been successful for increasing vaccination coverage in some circumstances. However, not enough evidence was available to draw firm conclusions about effectiveness, or to recommend widespread implementation. There was agreement with this in the qualitative research. Both parents and professionals recognised that financial incentives could be effective in some circumstances. Parents living in deprived circumstances were particularly identified as being potentially responsive to financial incentives.

The DCE found that parents preferred financial rewards with higher values. In contrast, whilst parents in the focus groups were not asked to agree on a specific appropriate level of incentive, they often felt that even £50 (~US$72; €66) was too high. Despite this, the survey identified that 80% of those who would not require a financial reward to vaccinate their children would still accept one if it was offered. Thus, whilst there may be a general perception that gaining financial rewards should not be the appropriate motivation for vaccination (see below), this does not mean that people would not accept such rewards, or that they would not be effective in some cases. Indeed, around one quarter of survey respondents stated that they would require a financial reward to fully vaccinate their children—although this proportion was statistically significantly higher in those with characteristics associated with incomplete vaccination (31%), than those without (19%).

The recognition of effectiveness, or at least potential effectiveness, is important—and not just from an evidenced-based policy point of view. Previous research has confirmed that the acceptability of incentive interventions increases with stated effectiveness,[14] and perceived ineffectiveness may be one reason why such interventions are often regarded as unacceptable.[29]

The belief that financial incentives may be most effective in deprived groups is likely to relate to the relative impact such financial incentives may have on household finances across the socio-economic spectrum. Others have proposed that incentive interventions may be particularly acceptable when targeted at those in most financial need. But there is also some concern that incentives may be most coercive in those who are least able to refuse the reward, due to financial pressures.[13] Whilst a number of outcome trials have focused particularly on deprived groups,[30, 31] there is an overall absence of evidence on whether effectiveness varies by socio-economic position.[8]

### Relative preferences for different interventions

There was a consistent finding from the systematic review and both components of the qualitative study that quasi-mandatory interventions were more acceptable than parental financial incentives. The qualitative study found an overall order of preference of: quasi-mandatory>universal financial reward>targeted financial reward. In contrast, the survey found an overall order of preference of: universal financial reward>quasi-mandatory interventions>targeted financial reward. A distinction between universal and targeted rewards could not be made in the systematic review.

The consistent preference for universal, compared to targeted, financial rewards appears to be related to issues of equity. The qualitative study identified that there was a general belief amongst participants that all health interventions should be available to all. The idea that parents who had delayed vaccination would become eligible for a financial reward under the targeted scenario was considered particularly inequitable and interpreted as rewarding ‘bad’ behaviour. Respondents were also concerned that such an intervention might lead to ‘gaming’, with parents deliberately delaying vaccinations in order to become eligible for the reward.

The concern for equity could be interpreted as contradicting the above finding in relation to differential effectiveness according to socio-economic position. However, whilst participants recognised that incentives may be more effective in some groups, this did not mean that they felt incentives should only be offered to those groups. It is possible that this finding is unique to the UK context where healthcare services are universally available to all.[13]

Apprehension about ‘gaming’ health promoting financial incentive interventions is frequently expressed in the literature.[13, 29, 32] Whilst there is little evidence of widespread ‘gaming’ from intervention trials,[33, 34] the concern that it might occur contributes to negative perceptions of these interventions. Further research is certainly needed to explore the extent and nature of any ‘gaming’, how this can be minimised, and how the limited gaming that appears to occur in practice can be adequately managed to quell public concerns.

The difference in relative preferences for universal incentives compared to quasi-mandatory interventions found between the qualitative study and the survey may reflect differences in the populations studied, the way questions were asked, or the setting in which preferences were elicited. The socio-economic profile of participants in the survey (almost 50% had completed degree-level education) was likely to be more affluent than participants in the qualitative study (educational attainment was not recorded, but parents were recruited mostly from Sure Start Children’s Centres which tend to serve more deprived communities). The survey was conducted anonymously online. In contrast, qualitative data collection took place in a social context with an interviewer and, in the case of focus groups, other participants, present. It is possible that universal incentives may be more acceptable than qualitative data suggests, but that people find it difficult to express this in social contexts. This could be interpreted as a form of ‘social desirability’ bias, where participants report what they feel is the socially acceptable answer in the context, rather than their ‘true’ beliefs and attitudes. Alternatively, participants in the qualitative studies often spent an hour or more discussing interventions, compared to the relatively quick online survey. Further, research is required to gain further clarity on why different results were found using different study designs.

### Cost and cost-effectiveness

Participants in the qualitative study expressed concern about the cost of financial incentives and queried whether resources might be more efficiently used in other ways. Whilst cost-effectiveness was not explicitly referred to, concerns about cost and efficiency certainly reflect this concept. In contrast, whilst quasi-mandatory interventions would also require substantial resources to develop and implement, the cost and cost-effectiveness of these interventions were not raised by participants. This may be because it was assumed that the tasks involved could be absorbed within the existing roles of staff working in education or child health settings.

Concerns about cost were not explicitly sought in the survey or embedded DCE. However, the survey questions did identify that the minimum effective incentive value amongst the minority of parents who stated they would require a financial incentive to fully vaccinate their children (25%) was around £110 (~US$159; €147). Most parents who would not require a financial reward to vaccinate, would still accept one (80%). The maximum acceptable level amongst these parents was around £70 (~US$101; €93).

Cost-effectiveness may be particularly salient when considering financial incentives because of the overt financial nature of the intervention.[29] The qualitative study and DCE were conducted in the UK, where the public is used to healthcare being funded through taxation and free at the point of delivery. However, the research was also undertaken during a period of economic austerity when questions were being raised about the sustainability of such a system. These contextual factors may have particularly increased concerns about whether or not such interventions would be affordable in the current economic climate.

As identified in the systematic review, the cost-effectiveness of both financial incentives and quasi-mandatory interventions for preschool vaccinations has not been well studied and is not yet known. However, previous research indicates that the great majority of public health interventions meet national criteria for cost-effectiveness used in England and Wales.[35]

### Alternative approaches to encouraging uptake of preschool vaccination

Participants in both components of the qualitative study made a variety of suggestions for alternative methods of increasing uptake of preschool vaccinations. These suggestions were spontaneous and unprompted, but common. In particular, both groups of participants suggested more flexibility in the timing and location of where vaccinations were delivered and improving the accessibility of information and education about vaccinations and vaccine-preventable diseases.

A preference for greater flexibility in appointments was also expressed in the DCE, where provision of out-of-hours appointments was preferred, particularly in those *without* characteristics associated with incomplete vaccination. Shorter waiting times were preferred, particularly in those *with* characteristics associated with incomplete vaccination. Reducing waiting times during normal clinic hours may, therefore, be particularly important for increasing vaccination uptake. Providing extended hours appointments would certainly be preferred by many parents, but would not be particularly attractive to those who are currently at risk of incompletely vaccinating their children and so may be of lower priority. One particular approach to avoid, identified in the qualitative study, was ‘block’ appointments where a group of parents are all given the same appointment time and then seen on a first-come, first-served basis.

Whilst the qualitative study found a general preference for wider availability of vaccinations, the DCE revealed that vaccinations provided by practice nurses in primary care settings were preferred to vaccinations provided by pharmacists, or by community nurses in mobile buses. This suggests that any changes to vaccination personnel and location would have to be carefully considered. Professionals in the qualitative study also raised considerable concerns about how data on vaccination status could be shared between those working in different sectors if the system were to be changed to enable different professional groups to deliver vaccinations.

Parents in the qualitative study showed an interest in vaccination delivery in children’s centres. In the DCE, preferences for vaccination delivery in children’s centres did not differ from those for practice nurses delivering vaccinations in primary care settings. This apparently contradictory finding could relate to the fact that many parents in the qualitative study were recruited through children’s centres and so were particularly familiar with this setting.

Whilst participants in the qualitative study acknowledged that substantial information on vaccinations is currently provided to new parents, there was widespread recognition that this was not provided in a format that parents found particularly accessible. The DCE found a preference for information about the risks and benefits of vaccinations to be provided in numerical format, rather than in charts and pictures, particularly in those parents with characteristics associated with incomplete vaccination. Presenting information in a range of different formats, and being sensitive to the different information needs of different parents, may help all parents feel their information needs are met.

## Discussion

### Summary of findings

We used Triangulation Protocol to integrate and synthesise findings from three different studies on the acceptability of parental financial incentives and quasi-mandatory interventions for preschool vaccinations. This is the first work we are aware of which draws together multi-methods results on acceptability of financial incentive interventions in any context.

There was a consistent recognition that incentives and quasi-mandatory interventions could be effective, particularly in more disadvantaged groups. Universal incentives were consistently preferred to targeted ones, but relative preferences for quasi-mandatory interventions and universal incentives varied between studies. The qualitative work revealed a consistent belief that financial rewards were not considered an appropriate motivation for vaccinating children. As incentives are designed to provide alternative, external, motivation for behaviours,[36] this may be an insurmountable barrier to widespread adoption of financial incentives for vaccination, or health behaviours more widely. The costs of financial incentive interventions appeared particularly salient and there were consistent concerns that incentives did not represent the best use of resources for promoting preschool vaccinations. Various suggestions for improving delivery of the current vaccination programme as an alternative to incentives and quasi-mandates were made, reinforcing a general negative view towards such interventions, despites the potential benefits also recognised.

### Strengths and limitations of methods

The complex and problematic nature of triangulation and integration, and the absence of detailed information on how to perform them, has been identified by a number of authors.[20, 37] Using the established framework of Triangulation Protocol lends rigour to our approach, by providing a clear structure for what we did and how.

Drawing on all four different types of triangulation—methodological, data, investigator and theoretical—increases the validity and reliability of our findings. It is unlikely that our results are due to a reliance on any single method, study population, researcher or theoretical perspective.

Although our systematic review was inclusive of studies from all high-income countries, the qualitative study and survey were conducted in England. The findings may not, therefore, be transferable to other contexts. In particular, there is some evidence that financial incentives for health behaviours are more acceptable in contexts without universal healthcare systems where the concept of paying for healthcare is more commonplace.[13]

Previous work has highlighted that the acceptability of structural public health interventions increases after implementation as people become familiar with the intervention and its practical implications.[23] It is possible that the generally low acceptability of parental incentives and quasi-mandatory interventions for preschool vaccinations described here reflects unfamiliarity with, lack of extensive public debate on, and lack of practical experience with such interventions. That is: a general fear of the unknown. Thus, the majority of our findings reflect the current situation in England, but it should not be assumed that this situation is necessarily immutable.

## Conclusions

The findings from this multi-methods programme of work indicate that financial incentives and quasi-mandatory interventions for increasing uptake of preschool vaccinations do not currently attract widespread enthusiastic support in the UK, although potential benefits were also recognised.

Acceptability was influenced by a general concern for equity and cost-effectiveness that may be particular to the current, UK context of a universal healthcare system in a time of austerity. Whilst there was some recognition that these interventions could be effective in some population groups, a number of other methods for increasing uptake of preschool vaccinations were proposed as currently being more effective and acceptable.
